# Hepatocyte Growth Factor Signaling in Intrapancreatic Ductal Cells Drives Pancreatic Morphogenesis

**DOI:** 10.1371/journal.pgen.1003650

**Published:** 2013-07-25

**Authors:** Ryan M. Anderson, Marion Delous, Justin A. Bosch, Lihua Ye, Morgan A. Robertson, Daniel Hesselson, Didier Y. R. Stainier

**Affiliations:** 1 Department of Biochemistry and Biophysics, Programs in Developmental and Stem Cell Biology, Genetics and Human Genetics, Liver Center and Diabetes Center, University of California San Francisco, San Francisco, California, United States of America; 2 Herman B. Wells Center for Pediatric Research, Department of Pediatrics, Indiana University School of Medicine, Indianapolis, Indiana, United States of America; University of Pennsylvania School of Medicine, United States of America

## Abstract

In a forward genetic screen for regulators of pancreas development in zebrafish, we identified *donut^s908^*, a mutant which exhibits failed outgrowth of the exocrine pancreas. The *s908* mutation leads to a leucine to arginine substitution in the ectodomain of the hepatocyte growth factor (HGF) tyrosine kinase receptor, Met. This missense mutation impedes the proteolytic maturation of the receptor, its trafficking to the plasma membrane, and diminishes the phospho-activation of its kinase domain. Interestingly, during pancreatogenesis, *met* and its *hgf* ligands are expressed in pancreatic epithelia and mesenchyme, respectively. Although Met signaling elicits mitogenic and migratory responses in varied contexts, normal proliferation rates in *donut* mutant pancreata together with dysmorphic, mislocalized ductal cells suggest that *met* primarily functions motogenically in pancreatic tail formation. Treatment with PI3K and STAT3 inhibitors, but not with MAPK inhibitors, phenocopies the *donut* pancreatic defect, further indicating that Met signals through migratory pathways during pancreas development. Chimera analyses showed that Met-deficient cells were excluded from the duct, but not acinar, compartment in the pancreatic tail. Conversely, wild-type intrapancreatic duct and “tip cells” at the leading edge of the growing pancreas rescued the *donut* phenotype. Altogether, these results reveal a novel and essential role for HGF signaling in the intrapancreatic ducts during exocrine morphogenesis.

## Author Summary

The pancreas functions as an endocrine and exocrine gland that secretes hormones regulating blood glucose homeostasis, and pancreatic juice that aids the digestion and absorption of nutrients, respectively. Contrary to endocrine tissue development, that of the exocrine pancreas has received less attention. We conducted a forward genetic screen in zebrafish and identified HGF/Met signaling as a key regulator of exocrine development. We called the mutant *donut* because the body of the pancreas fails to elongate and thus remains rounded. The mutation leading to this phenotype affects the extracellular domain of Met, the hepatocyte growth factor (HGF) receptor, impairing its maturation, plasma membrane localization and phospho-activation. Although HGF/Met signaling may elicit many context-dependant cellular responses, our data indicate that HGF/Met signaling triggers the migration, but not the proliferation, of the pancreatic ductal cells to drive the extension of the pancreatic tail.

## Introduction

The vertebrate pancreas is an endodermal organ that is part endocrine, releasing hormones that regulate glucose metabolism, and part exocrine, releasing pancreatic juices that aid in digestion. Pancreatic endocrine and exocrine developmental dysmorphogenesis and dysregulation, including diabetes mellitus and pancreatic adenocarcinoma, can result in human diseases with high morbidity and mortality. Thus, a more sophisticated understanding of molecular mechanisms mediating pancreatic development and homeostasis will certainly refine the treatment of these diseases.

In zebrafish as in mammals, all pancreatic endocrine and exocrine tissues derive from the fusion of a dorsal and ventral bud arising at the level of somites 2–9 [1], [2], [3]. In zebrafish, the dorsal bud generates the principal islet by 24 hours post fertilization (hpf), and fuses with the emerging ventral bud between 40–44 hpf [4], [5]. Around 52 hpf, acinar and ductal cells start to expand caudally to form the tail of the pancreas [5], [6], [7]. The pancreatic mesenchyme is essential for the induction, growth, branching, and cytodifferentiation of the pancreatic epithelium [8]. While several mesenchymal signals mediating pancreatic induction have been identified (reviewed in [9]), our knowledge of how the mesenchymal/epithelial signaling pathways regulate pancreatic growth and branching is more limited [8].

Hepatocyte Growth Factor (HGF) is a stromally-produced ligand which binds Met, a receptor tyrosine kinase that is predominantly expressed in epithelia. Upon receptor dimerization and autophosphorylation, Met activates a bevy of cellular processes including motogenesis, tubulogenesis, mitosis, chemotaxis, and cell survival [10]. During organogenesis, HGF/Met signaling has been shown to be involved in liver and placenta formation, as well as in the migration of hypaxial muscle precursors into limbs [11], [12], [13], [14]. However, the role of HGF/Met signaling in vertebrate pancreas development remains unclear. Both HGF and Met are expressed in the developing rodent pancreas [15], [16], but pancreatic phenotypes have not been characterized in global knockout mice. Studies have been mostly focused on the role of HGF/Met signaling in pancreatic tumorigenesis and beta-cell survival. Indeed, pancreas-specific Met knockout mice are euglycemic and morphologically unaffected at maturity, but show impaired beta-cell homeostasis during pregnancy [17] and following STZ-induced islet inflammation [18]. Even though HGF/Met signaling has been shown to activate the PI3K/Akt and ERK pathways in acinar cells [19], its biological role during exocrine pancreas development remains undetermined.

## Results/Discussion

### Identification and genetic mapping of *donut^s908^* mutants

**Figure 1:**
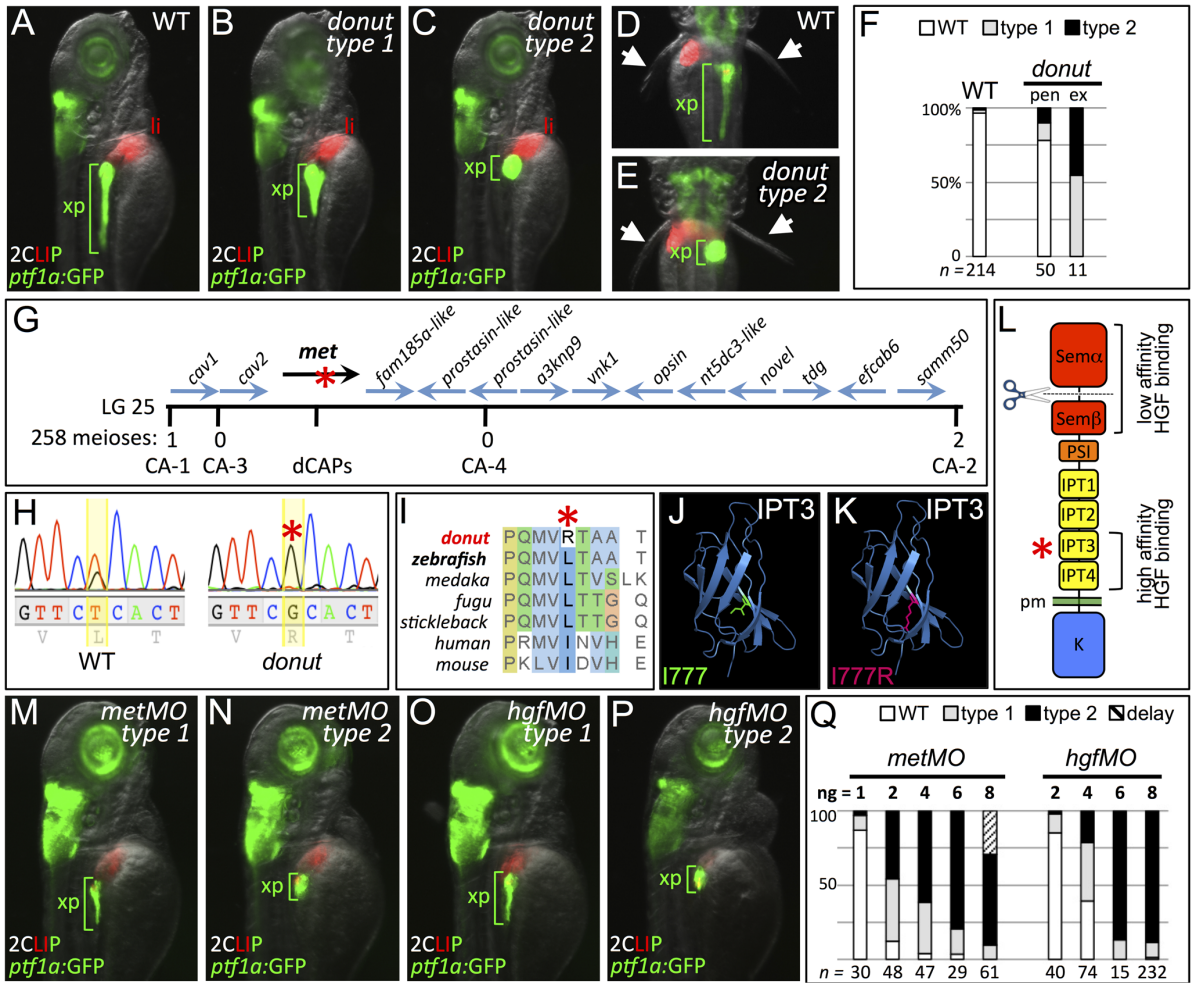
*donut^s908^* is a hypomorphic allele of *met*. (A–E) Exocrine pancreas (xp) structure marked by *ptf1a:*GFP expression in 3 dpf WT (A,D), and in type 1 (B) and type 2 (C,E) *donut* mutants. The pectoral fins (arrowheads) of *donut* mutants lack muscle tone, often asymmetrically, leading to their “open wing” appearance (right arrow, E). (F) Penetrance (pen) and expressivity (ex) of the *donut* phenotype. A small fraction of WT embryos shows *donut*-like pancreatic phenotypes, while 22% of clutchmates from heterozygous intercrosses exhibited either a spherical (55%) or an intermediately shortened (45%) pancreas. *n* below bars represents the number of embryos examined from WT clutches (right), heterozygote in-crossed clutches (center), and embryos exhibiting donut-like pancreatic phenotypes (right). (G–I) *donut* mutants have a lesion in *met. donut* was mapped to a critical interval on Chr. 25 containing 14 annotated genes (G); *met* showed a T2324G variant (H) causing an L775R amino acid substitution (I). (J,K) Model structures of the human MET IPT3 domain with isoleucine 777 (analogous to zebrafish residue L775) marked green (J) or substituted arginine marked red (K). (L) Diagram of Met showing the I777R substitution localized to the high affinity HGF binding site in IPT3, and the furin cleavage site in the semaphorin-like domain. (M–P) Morpholino-mediated knockdown of *met* (M,N) or *hgfa/b* (O,P) resulted in phenocopy of the *donut* mutation. (Q) Dose dependence of morpholino-induced phenotypes. At 8 ng, metMO exhibited some non-specific developmental delay, suggesting toxicity. pen, penetrance; ex, expressivity; li, liver; pm, plasma membrane.

To find novel regulators of endodermal organ morphogenesis and differentiation, we conducted a forward genetic screen utilizing doubly transgenic (2CLIP: 2-color liver, insulin, exocrine pancreas) zebrafish with EGFP-expressing pancreatic acinar cells and DsRed-expressing pancreatic beta-cells and hepatocytes [20]. Using this approach, we identified *donut^s908^* mutants, which show shortened (type 1) or absent (type 2) exocrine pancreatic (xp) tails. *Tg(ptf1a:GFP)^jh1^* [21] was crossed into the *donut^s908^* background to reveal the short exocrine pancreas phenotype at 3 days post fertilization (dpf; Fig. 1A–E), prior to the terminal differentiation of acinar cells. Differentiation of the principal islet (beta-cells, β), acinar and ductal cells, and liver (li) appeared unaffected in *donut* mutants (Fig. 1B,C; and see below). Nearly 25% of progeny from heterozygous intercrosses showed the *donut* phenotype, evenly split between types 1 and 2 (Fig. 1F). The variable expressivity suggested either a hypomorphic mutation in *donut*, and/or a genetic interaction with other loci.

To isolate the molecular lesion responsible for the *donut* phenotype, we used bulk segregant analysis and localized *donut* on linkage group 25; we then used z-markers and customized CA-repeat markers to define a critical interval containing 14 candidate genes (Fig. 1G). In sequencing these candidates, we identified a T2324G variant in the coding sequence of the *met* gene, resulting in an L775R missense substitution (Fig. 1H). The Met^L775R^ substitution in *donut^s908^* mutants represents a significant shift in amino acid charge and polarity at a residue that is conserved in vertebrates as a leucine or an isoleucine (Fig. 1I). Importantly, L775 is localized to the IPT3 (immunoglobulin-like regions in plexins and transcription factors 3) domain in the Met ectodomain, which together with IPT4 comprises a high affinity HGF binding site (Fig. 1L) [22], [23]. Moreover, protein domain modeling based on human MET IPT3 indicates that the analogous residue, I777, resides in a solvent inaccessible region of IPT3, and that its substitution with arginine may be sterically unfavorable (Fig. 1J,K), suggesting that Met folding may be compromised in zebrafish *donut* mutants.

We hypothesized that the *donut^s908^* mutation would result in a Met loss of function. To test this hypothesis, we injected zygotes with translation-blocking *met* morpholinos (*metMO*). We found that even low doses of *metMO* (2 ng) could reproduce the shortened pancreatic tail phenotype observed in *donut* mutants with high penetrance (Fig. 1M,N). Importantly, when *donut^s908+/−^* embryos were injected with a near-threshold (1 ng) dose of MO, 53.4% displayed a short pancreas phenotype, as compared to 29.1% of injected wild-type embryos (Fig. S1). Since HGF is the only demonstrated ligand for Met, we reasoned that knockdown of both zebrafish *hgf* paralogs, *hgfa* and *hgfb*, should also mimic *donut* mutant phenotypes. Similar to *donut* mutants, we found that embryos injected with a mixture of MOs directed against *hgfa* and *hgfb* (*hgfMO*) also developed shortened pancreata (Fig. 1O,P). We observed a positive correlation between the injected quantity of each morpholino and the penetrance and expressivity of the pancreatic tail phenotypes (Fig. 1Q), with expressivity variation resolving towards the more severe type 2 phenotype at higher doses. Finally, *donut* mutants frequently lack muscle tone in the pectoral fins, suggesting a failed morphogenesis of hypaxial muscle (Fig. 1D,E, arrowheads; Fig. S2), which corroborates previous studies of Met function in myogenic precursor cells in mouse and zebrafish [11], [12]. In sum, these data are consistent with the interpretation that the phenotypic spectrum observed in *donut^s908^* mutants is due to a hypomorphic effect of the L775R substitution.

**Figure 2:**
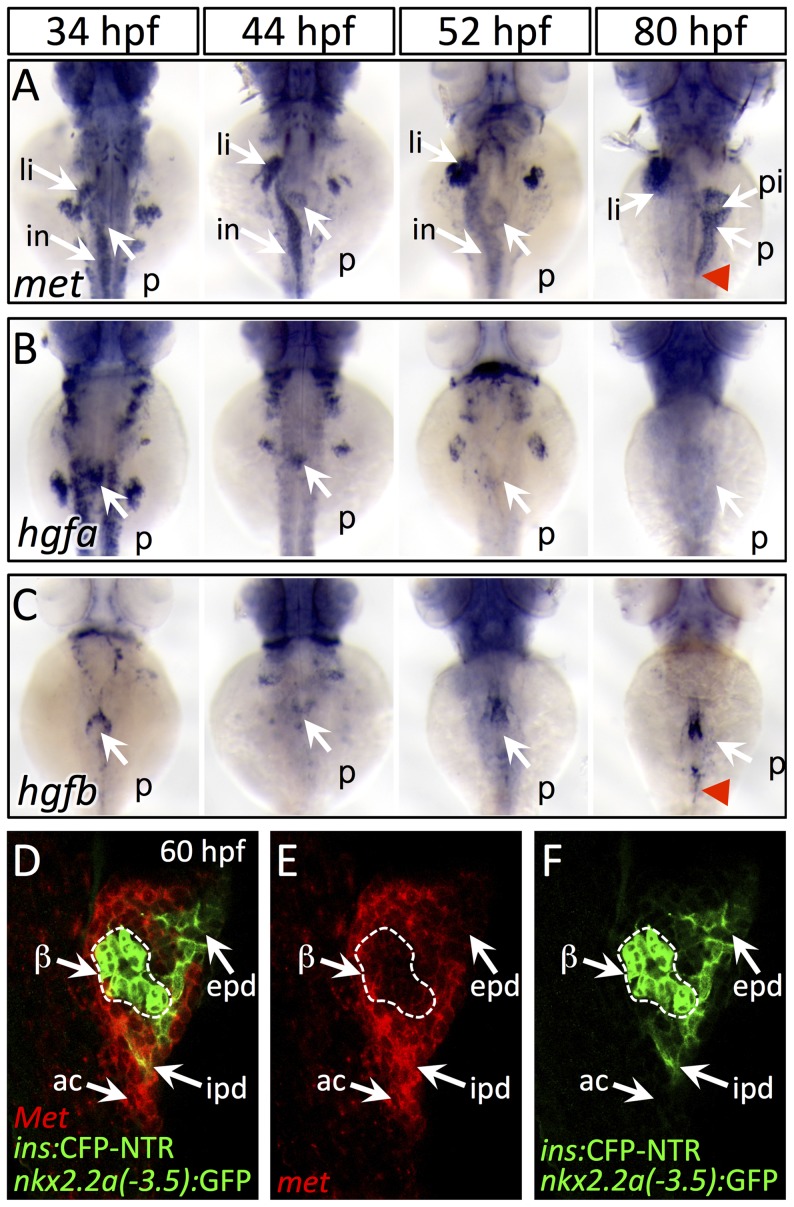
Dynamic expression of *met* and *hgf* during pancreas morphogenesis. (A) *met* is expressed strongly in the pancreatic bud (p), liver bud (li), and intestinal bulb (in) from 34–52 hpf. At 80 hpf, *met* expression is found throughout the exocrine pancreas and is diminished in the principal islet (pi). (B,C) *hgfa* (B) and *hgfb* (C) are expressed at 34–44 hpf near the dorsal pancreatic bud prior to pancreatic tail formation. At 52 hpf, at the onset of pancreatic tail formation, both *hgfa* and *hgfb* are expressed near the distal tip of the pancreatic bud, with *hgfb* expression persisting until the completion of pancreatic tail outgrowth (red arrowhead). (D–F) One-micron confocal plane images of *duct:GFP;ins:CFP-NTR* embryos stained for *met* mRNA using fluorescent *in situ* hybridization shows *met* expression (red) in both acinar cells (ac) and intrapancreatic ducts (ipd; green), but not in the principal islet (green outlined in white). Dorsal views, anterior to the top. beta-cells, β; epd, extrapancreatic duct.

To clarify how HGF signaling is implemented in pancreatogenesis, we examined the expression of *met*, *hgfa* and *hgfb* during several stages of pancreas formation. We also analyzed the expression of both *furin* genes, *furina* and *furinb*, which encode the proteases that cleave Met into a mature form. At 34 hpf, which is prior to pancreatic bud fusion, *met* is expressed throughout the endodermal organ forming region, including pancreas, liver, and intestine anlagen (Fig. 2A). Additionally, *met* expression is detected in pancreatic endoderm before (44 hpf), during (52 hpf), and after its caudal extension (80 hpf; Fig. 2A). We found that *hgfa* and *hgfb* were expressed adjacent to the dorsal pancreatic bud at 34 hpf, and that their expression remains in register with the leading edge of the growing pancreatic tail (red arrowhead) between 52 and 80 hpf (Fig. 2B,C). Importantly, both *furina* and *furinb* are expressed in endodermal tissue throughout pancreatogenesis, with *furina* present in liver and pancreas at 75 hpf (Fig. S3), indicating that the process of Met maturation is active in these organs. To determine which exocrine tissues express *met* during pancreatic tail morphogenesis, we performed fluorescent *in situ* hybridization in the *Tg(nkx2.2a(-3.5 kb):GFP)* line (hereafter *duct:GFP*) which highlights the intrapancreatic ducts (IPDs) [6]. We found that within the pancreatic tail, *met* was expressed in both IPD and acinar cells at 60 hpf, a time of active pancreatic tail extension (Fig. 2D–F). A role for Met signaling in pancreatic exocrine morphogenesis has not been reported in mammalian model systems. *Hgf* and *Met* knockout mice die during organogenesis precluding analysis [11], [13], [14], and a pancreas specific knockout (PancMet KO) showed no clear morphological defect of the adult pancreas [18]. However, as pancreas development was not described in PancMet KO, it is possible that a transient phenotype or developmental delay could have been overlooked. Furthermore, it is likely that early mosaic recombinase activity inherent to the *Pdx1:Cre* line that was utilized in these experiments leaves a population of wild-type cells in these mice. These cells may be competent to effect normal morphogenesis, and would mirror what we observed in chimera studies (see below). Alternately, residual Met protein, which is expressed in endoderm prior to the onset of *Pdx1* expression, or a parallel/redundant mechanism could drive exocrine outgrowth in mammals. Additional targeted studies are needed in mice to determine whether the role of Met in exocrine pancreas growth is conserved.

### Met^L775R^ shows impaired plasma membrane targeting and activation

**Figure 3:**
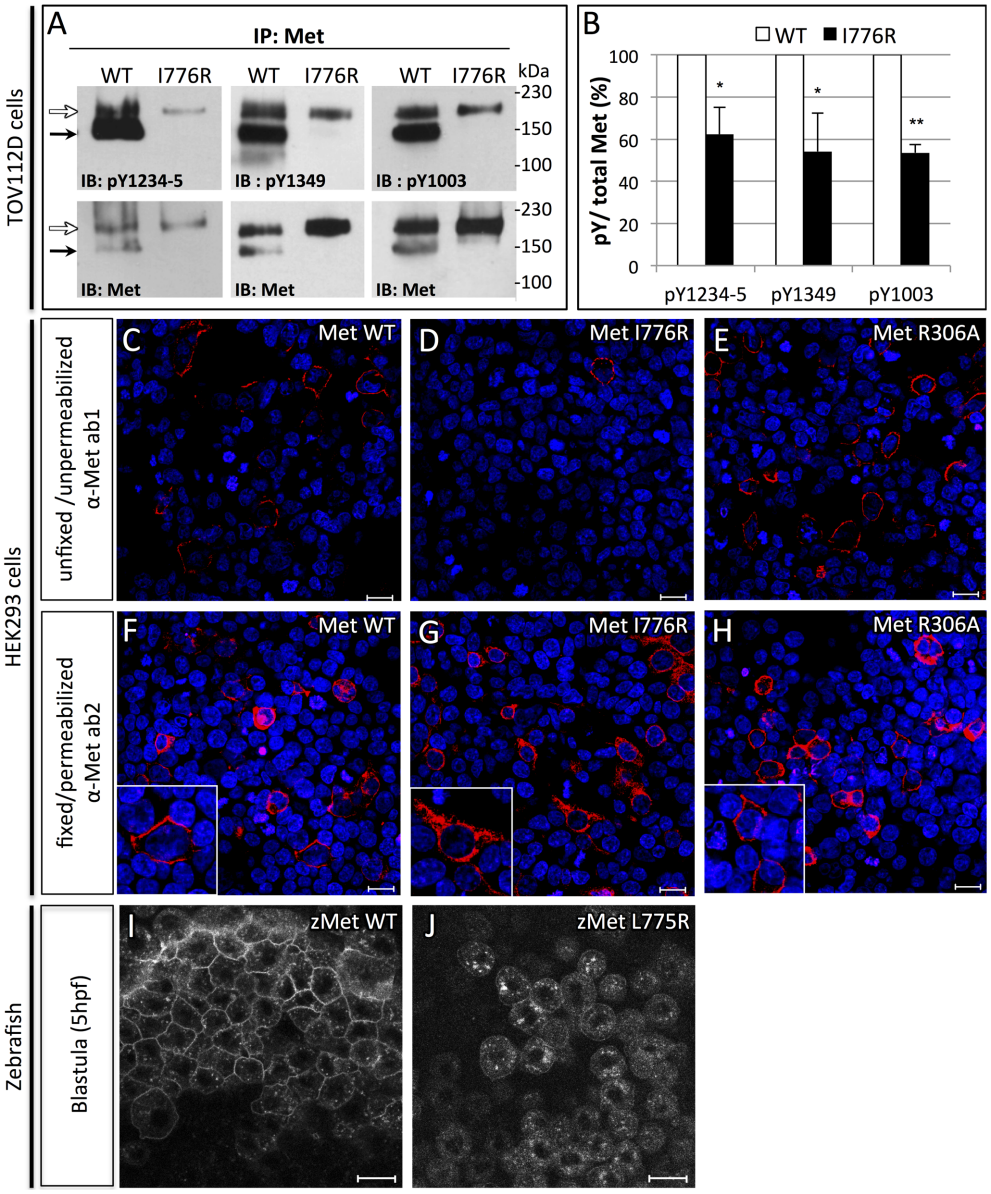
Orthologous murine *donut* mutation, I776R, impairs HGF signaling and receptor trafficking. (A) Immunoblots of Met protein prepared from lysates of TOV112D cells transfected with murine *Met^WT^* or *Met^I776R^*. Upper panels blotted with anti-pY show diminished phosphorylation at tyrosines critical for signaling and lower panels blotted with anti-Met show loading controls. Precursor (white arrows) and mature (black arrows) forms of Met^WT^ are both detected, but Met^I776R^ is only present in the precursor form. (B) Quantification by densitometry of phosphorylated Met *versus* total Met in panel A (t test, * p<0,05; **p<0,01). (C–H) Immunofluorescence staining of HEK293 cells transfected with *Met^WT^* (C,F), *Met^I776R^* (D,G), or furin cleavage-incompetent *Met^R306A^* (E,H). Unfixed, unpermeabilized cells were stained with anti-Met ab1, which binds an extracellular epitope (C–E), and fixed, permeabilized cells were stained with ab2, which binds an intracellular epitope (F–H). Met^WT^ and Met^R306A^ are localized to the plasma membrane (C, E, insets in F, H), but Met^I776R^ is not (D), and is mostly retained in the cytoplasm (G, inset). Intracellular staining of Met demonstrates similar transfection efficiency. DAPI staining (blue) marks nuclei. (I–J) Live imaging of zebrafish blastulae injected with zebrafish Met^WT^-mCherry (I) and Met^L775R^-mCherry (J) at 5 hpf. Zebrafish Met^L775R^-mCherry largely fails to localize to the plasma membrane as compared to Met^WT^. Scale bars, 20 µm.

To model how the *donut* mutation affects Met function, we generated an I776R point mutation in murine Met, an analogous lesion to the *donut^s908^* mutation, and then transfected either *Met^WT^* or *Met^I776R^* into TOV112D cells. This cell line lacks endogenous Met activity, but expresses the intracellular components of the Met signaling pathway [22], [24]. Met is translated as a single polypeptide chain which is cleaved by furin into alpha and beta chains [25]. In TOV112D cells transfected with Met^WT^, most of the nascent polypeptide was cleaved to the mature form (150 kDa+30 kDa), while a small proportion of the receptor remained unprocessed (190 kDa). Since the I776R lesion lies far from the furin cleavage site (residues 302–307) [26], we were surprised to find that the Met^I776R^ precursor polypeptide failed to be cleaved (Fig. 3A, bottom panels). Since cleavage-incompetent Met mutants (i.e. Met^R306A^) can signal normally [27], we next assessed the signaling efficacy of Met^WT^ and Met^I776R^ using specific anti-phosphorylated Met antibodies directed against several key tyrosine residues. Upon HGF binding, Met dimerizes and its cytoplasmic catalytic region is activated by trans-phosphorylation of tyrosines Y1234 and Y1235. Subsequently, residues Y1349 and Y1356 are phosphorylated in the Met multifunctional docking site, which connects Met to multiple downstream branches, including Ras/MAPK and PI3K, through several adapter proteins. In addition, phosphorylation of tyrosine Y1003 negatively regulates Met signaling by promoting receptor turnover. Compared to Met^WT^, phosphorylation of Y1234-5, Y1349, and Y1003 were significantly diminished in Met^I776R^ (Fig. 3A,B). Finally, since cleavage by Furin occurs in the Golgi apparatus [28], we hypothesized that intracellular trafficking of Met^I776R^ from the endoplasmic reticulum to the plasma membrane was impaired. To investigate this hypothesis, we next examined the presentation of Met at the plasma membrane using an antibody that binds specifically to the Met ectodomain (α-Met ab1). Antibody staining was evident at the membrane of unfixed, unpermeabilized HEK293T cells expressing Met^WT^ or cleavage incompetent Met^R306A^, but not Met^I776R^ (Fig. 3C–E). However, all of these Met variants were detected in fixed and detergent-permeabilized cells using an antibody that recognizes an intracellular epitope (α-Met ab2), thereby indicating similar transfection efficiencies of the three constructs (Fig. 3F–H). Thus, these data suggest that Met^I776R^ is retained in an intracellular compartment, and that the lack of Met^I776R^ cleavage per se is not causing this retention. To further test this hypothesis, RNA encoding zebrafish Met^WT^ or Met^L775R^ fused to mCherry were injected in zebrafish embryos and the localization of the proteins analyzed at blastula stages (Fig. 3I–J). As observed in cell culture, Met^L775R^ was not detected at the plasma membrane *in vivo*. This defect is not simply a delay of membrane targeting, as similar results were observed at later time points (data not shown). Together, these data support the hypothesis that deficient signaling through Met^L775R^ causes the hypomorphic effect observed in *donut^s908^* mutants. Likely, reduced Met signaling is due to (1) localization of Met^L775R^ away from the plasma membrane, (2) impaired binding of HGF to the high-affinity binding site in IPT3-4, and/or (3) impaired assembly of Met co-receptor moieties, that would then lead to defective phospho-activation of Met.

### Cell migration but not proliferation is impaired by the *donut^s908^* mutation

**Figure 4:**
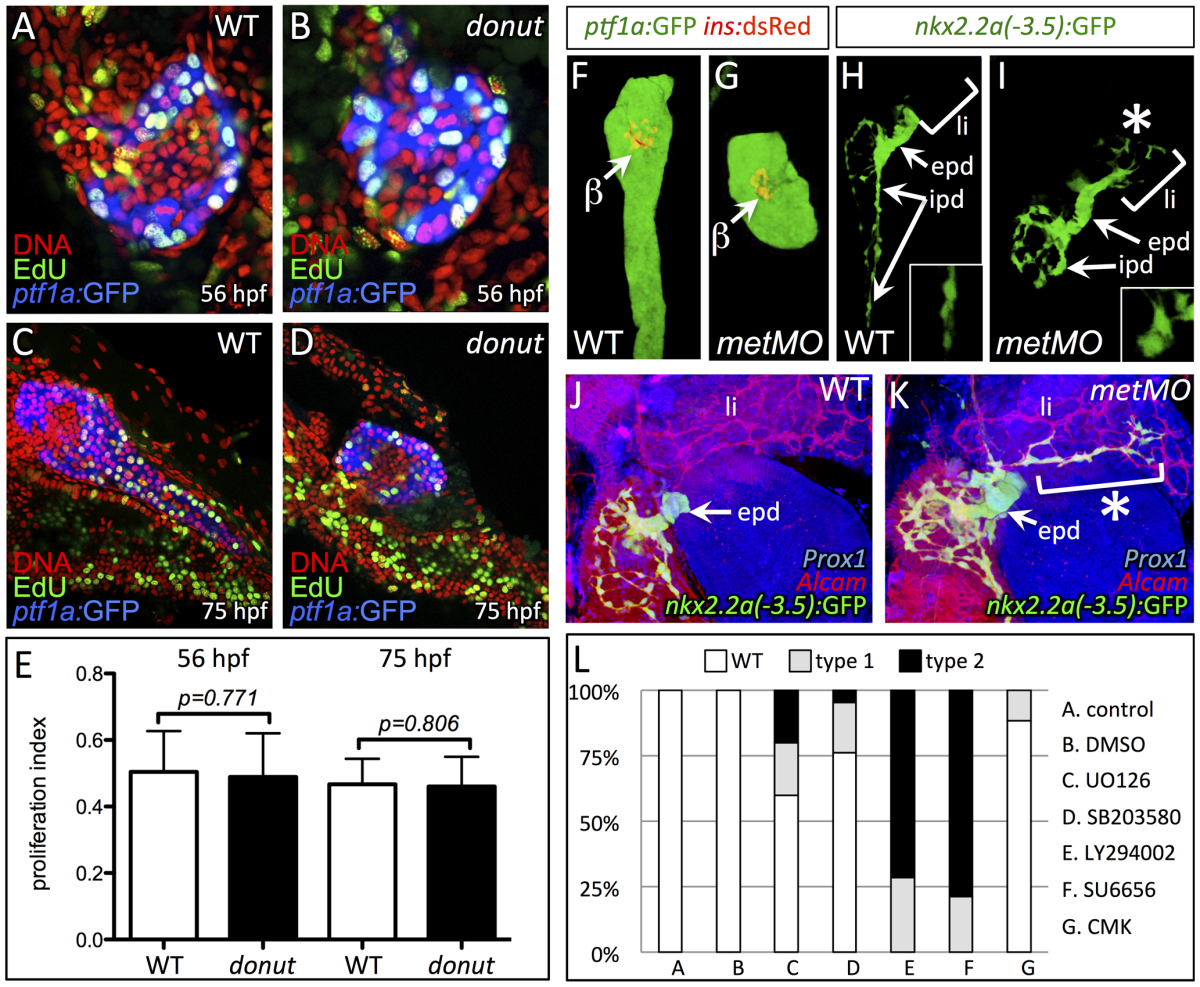
Cell migration, but not proliferation, is dysregulated in *donut^s908^* mutants. (A–D) EdU incorporation assay in WT (A,C) and *donut^s908^* mutant (B,D) animals at 56 (A,B) and 75 (C,D) hpf. (E) Quantification of proliferation assay data shows no significant change in acinar cell proliferation at early or late stages of pancreatic tail formation. (F–I) Morphology of exocrine tissues at 84 hpf in WT (F,H) and *metMO-*injected (G,I) larvae revealed by *Tg(ptf1a:*GFP) (acinar; F,G) or *Tg(nkx2.2a(-3.5 kb):*GFP) (duct; H,I) expression. In *metMO-*injected embryos, acinar and ductal cells fail to migrate caudally, and remain near the principal islet (β). In *metMO* larvae, ductal cells exhibit a more rounded morphology in the exocrine pancreas (insets) and are also observed in the liver region (asterisk). (J,K) 84 hpf WT (J) and *metMO*-injected (K) *Tg(duct:GFP)* larvae stained with Prox1 (blue) and Alcam (red). Pancreatic ductal cells are ectopically localized in the liver (li) along tracts of biliary ducts. (L) Quantification of pancreatic tail defects in small molecule treated larvae; inhibitors of the MAPK pathway (UO126, SB203580) and furin function (CMK) had little effect on pancreatic tail outgrowth, while inhibitors of PI3K (LY294002) and STAT3 (SU6656) function mimicked the type 1 and type 2 *donut* phenotypes. epd, extrapancreatic duct.

Growth and elongation of the pancreatic tail involves both proliferation and directed migration of exocrine cells [6], [7]. To analyze which mechanism is impaired in *donut* mutants, we first assayed cell proliferation using 30 min incorporation of the thymidine analog EdU at two time points during pancreatic tail growth: 56 and 75 hpf (Fig. 4A–D). Quantification of labeled cells showed no significant difference in exocrine cell proliferation between WT and *donut* mutant animals (Fig. 4E). To determine whether acinar or ductal cell migration was affected in *donut* mutants, we next examined the distribution of acinar and ductal cells using *ptf1a:GFP* and *duct:GFP*, respectively, in 84 hpf wild-type (WT) and *metMO*-injected (Fig. 4F–I) larvae. Although *ptf1a:GFP*+ acinar cells were always confined to the pancreas, *duct:GFP*+ cells were clearly localized in the hepatic region in both type 1 and type 2 mutants. Furthermore, while IPD cells were elongated and spindle-shaped in WT, they showed a more rounded morphology in *metMO*-injected larvae (Fig. 4H,I, insets). We marked hepatocytes and biliary ducts with Prox1 and Alcam antibodies, respectively, and found that *duct:GFP*+ pancreatic cells had extensively invaded the liver, tracking along the biliary duct network (Fig. 4J,K). Taken together, our data show that HGF/Met signaling promotes invasive cell behavior during pancreatic tail morphogenesis, rather than the proliferation of exocrine cells.

The invasive cell behaviors activated by HGF/Met signaling, such as motility and proliferation, work through multiple parallel downstream branches of Met signaling. Three key branches involve the PI3K, ERK, and STAT3 pathways, which may be activated by direct interaction with Met, or through adapter proteins: the PI3K/Akt pathway elicits cell migration via Rac1, as well as cell survival; the ERK branch promotes proliferation and transformation; and STAT3 signaling mediates branching morphogenesis and proliferation (reviewed in [10]). To dissect which specific branches might be critical for Met-mediated exocrine morphogenesis, we treated larvae during the formation of the pancreatic tail with specific inhibitors (Fig. 4L). Inhibition of ERK signaling with the MEK inhibitor UO126 or the p38 MAPK inhibitor SB203580 resulted in a mild phenocopy of *donut* type 1 and type 2 mutants, ranging from 25–40%. However, treatment with the potent PI3K inhibitor LY294002 or the STAT3/Src family kinase inhibitor SU6656 generated a nearly perfect phenocopy of type 1 and 2 *donut* mutants. These data confirm our previous observations that cell proliferation via MAPK/MEK is not the primary mechanism driving pancreatic tail outgrowth. The furin protease inhibitor CMK caused only a minor effect on pancreatic tail formation confirming that failure of proteolytic cleavage of Met may not cause the reduced signal transduction capacity of Met^L775R^.

### Met signaling is autonomously required in IPD cells and “tip cells” for pancreatic tail elongation

**Figure 5:**
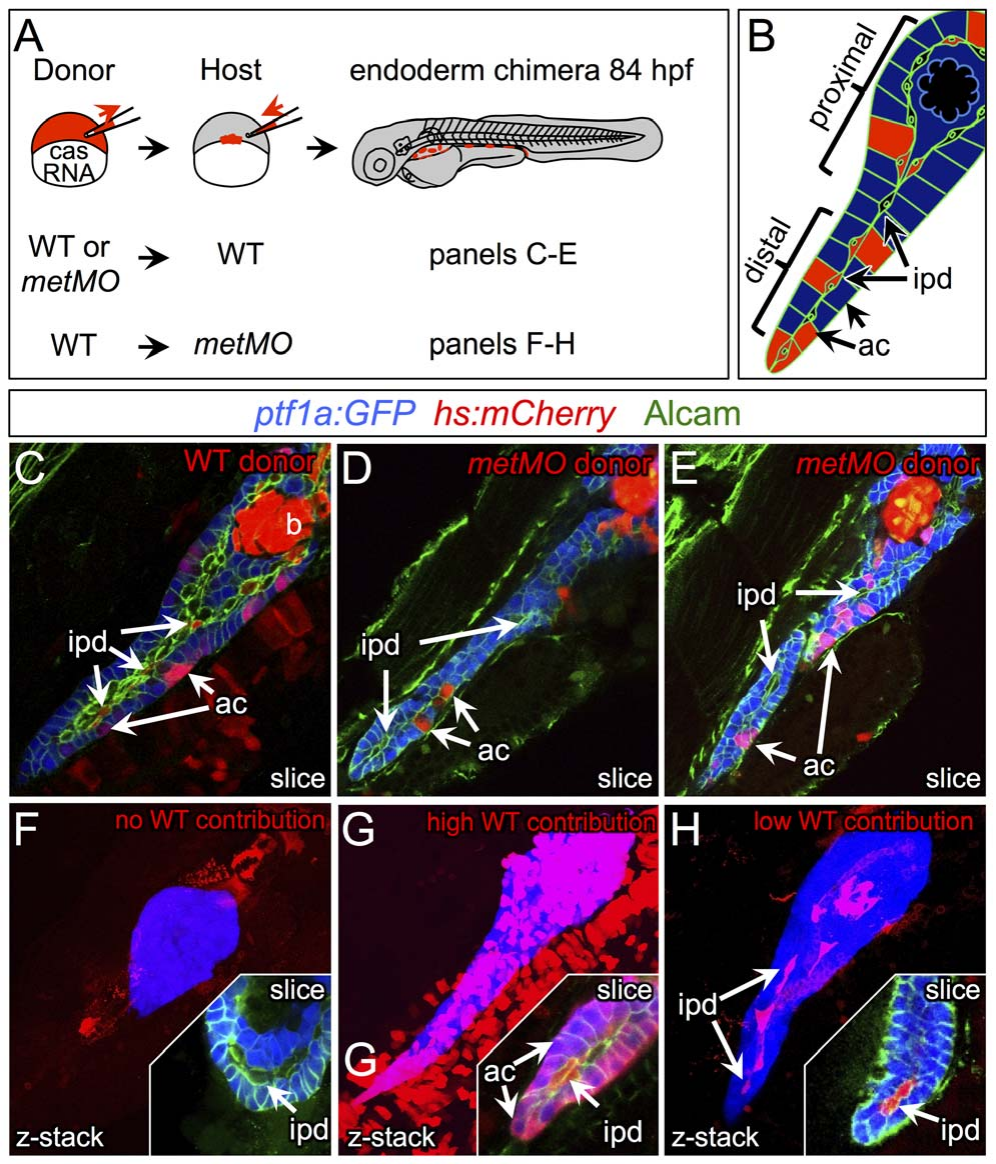
HGF/Met signaling is required in intrapancreatic ducts for pancreatic tail morphogenesis. (A) Schematic of cell transplantation experiments. WT or *metMO*-injected donor cells were transplanted into WT hosts (top), and WT donor cells were transplanted to *metMO*-injected hosts (bottom); *cas* mRNA injection biased donor cells toward endodermal differentiation. (B) Scheme used to identify and quantify contribution of transplanted cells in chimeras. Donor cells are marked by *Tg(hs:mCherry)* (red), acinar cells are marked by *Tg(ptf:GFP)^jh1^* (blue), and Alcam immunostaining (green) delineates the ducts. (C–E) Single plane confocal images of WT (C) and *metMO* (D,E) donor endoderm transplanted into WT hosts: WT donor cells contributed to both intrapancreatic ductal (ipd) and acinar (ac) compartments, but *metMO* donor cells were excluded from the ducts. (F–H) WT≫*metMO* hosts: With no WT contribution to the pancreas, *metMO* morphants show *donut*-like phenotypes (E); with high contribution of WT donor cells, the pancreatic tail growth is rescued (G), and the tip of the tail is almost entirely comprised of WT donor cells (inset of single confocal slice); with moderate contribution of WT donor cells, the pancreatic tail morphology can be rescued with only WT ductal cells (H), and no WT acinar cells are observed in the tip (inset of single confocal slice).

The differentiating exocrine pancreas is highly organized at the onset of pancreatic tail elongation [6], [7]. Acinar and ductal cells are the only two cell types found in the growing pancreatic tail, and they are always closely associated. Even though *met* is expressed in both cell types, we hypothesized that it was required primarily in ductal cells, since these cells were mislocalized and dysmorphic in *metMO*-injected larvae. To test this hypothesis, we performed cell transplantation experiments to generate chimeras and determine the autonomy of Met function in the pancreas (Fig. 5A). First, blastomeres isolated from control or *metMO*-injected *Tg(hs:mCherry)* (see Methods) embryos were directed toward an endodermal fate by *cas* mRNA coinjection [29], [30], and were transplanted into WT hosts. Incorporated donor cells ubiquitously express mCherry RFP upon heat shock induction, and were quantified based on their contribution to *ptf1a:GFP+* acinar or *ptf1a:GFP−* ductal cell compartments in the distal or proximal portion of the exocrine pancreas (Fig. 5B). In contrast to WT cells that contributed evenly to both acinar and ductal compartments, *metMO*-injected cells were strongly biased toward the acinar cell and proximal ductal compartments, and were largely excluded from the distal ductal compartment (Fig. 5C–E; Tables 1,2). We observed similar results when we transplanted endodermal cells from *donut* mutant embryos into *Tg(hs:mCherry)* hosts (data not shown). Next, we assessed the rescue of exocrine pancreatic tail outgrowth by transplanting varying numbers of WT *Tg(hs:mCherry)* cells into the endoderm of *metMO*-injected hosts (Tables 3,4). *metMO*-injected larvae lacking WT contribution showed a type 1 *donut* phenotype (Fig. 5F), whereas a high contribution of WT endoderm cells led to a complete rescue of the pancreatic tail outgrowth (Fig. 5G). Notably, the tip of the rescued pancreatic tail appeared to be composed exclusively of WT donor cells (Fig. 5G, inset). In larvae with fewer integrated WT cells, the pancreatic tail was also rescued, with the WT cells being preferentially found in the IPD (Fig. 5H, inset). Finally, we confirmed that WT endoderm could not similarly rescue the short exocrine pancreas phenotype observed in *hgfMO-*injected embryos, consistent with a mesenchyme-specific role for *hgfa* and *hgfb* (Fig. S4). These data indicate that endodermal Met signaling, probably preferentially in distal IPD cells, directs the caudal extension of the pancreatic tail.

**Table 1:** Endodermal contributions in WT>WT chimeric embryos.

donor contribution	prox. duct (*n*, %)	dist. duct (*n*, %)	prox. acinar *(n*, %)	dist. acinar (*n*, %)
high	*4/4*, 100%	*4/4*, 100%	*4/4*, 100%	*4/4*, 100%
low	*1/1*, 100%	*2/3*, 67%	*0/1*, 0%	*2/2*, 100%
total*	*5/5*, 100%	*6/7*, 86%	*4/5*, 80%	*6/6*, 100%

abbreviations: prox., proximal; dist., distal.

* Not every chimeric pancreas could be clearly assessed in all regions due to variations in mounting technique and limitations of confocal microscopy. Ambiguous samples were excluded from analysis.

**Table 2:** Endodermal contributions in *metMO*>WT chimeric embryos.

donor contribution	prox. duct (*n*, %)	dist. duct (*n*, %)	prox. acinar (*n*, %)	dist. acinar (*n*, %)
high	*4/10*, 40%	*2/10*, 20%	*9/10*, 90%	*8/10*, 80%
low	*7/14*, 50%	*2/14*, 14%	*12/14*, 86%	*12/14*, 86%
total	*11/24*, 46%	*4/24*, 17%	*21/24*, 88%	*20/24*, 83%

**Table 3:** Endodermal contributions in WT>*metMO*-injected chimeric embryos.

donor contribution	prox. duct (*n*, %)	dist. duct (*n*, %)	prox. acinar (*n*, %)	dist. acinar (*n*, %)
high	*15/16*, 94%	*16/18*, 89%	*16/16*, 100%	*18/18*, 100%
low	*4/7*, 57%	*7/9*, 78%	*1/7*, 14%	*1/9*, 11%
total*	*19/23*, 83%	*23/27*, 85%	*17/23*, 74%	*19/27*, 70%

**Table 4:** Breakdown of rescued embryos by WT tissue contribution.

total (*n*, %)	duct only (*n*, %)	acinar only (*n*, %)	duct & acinar (*n*, %)
*19/27*, 70%	*5/19*, 26%	*1/19*, 5%	*13/19*, 68%

We thus propose a model for pancreatic tail outgrowth in which ductal cells would respond to HGF secreted from the mesenchyme adjacent to the pancreatic primordium and initiate migration caudally. The HGF signal may constitute a chemotactic signal for the ductal cells, or simply a motogenic signal, with directionality imparted by distinct signaling pathways. Even though Met signaling appears to be required in IPD cells for pancreatic outgrowth, Met signaling may also be active in a small population of “tip” cells that are mature or differentiating acinar cells. From the analysis of a specific subset of chimeric embryos, ductal cells may thus be directing the migration of the pancreatic tail from a trailing position, possibly exhibiting cellular extensions, such as cytonemes or filipodia, that were not resolved in our studies.

Hence, our data expand the understanding of the role of HGF/Met signaling in mitogenic, motogenic and morphogenetic events required for the development, homeostasis and regeneration of different tissues. In addition, they provide a developmental framework to dissect the role of Met in pancreatic cancer stem cells [31], [32].

## Materials and Methods

### Zebrafish strains

Fish were raised and maintained under standard conditions [33]. Pigmentation was inhibited with 0.02 mM phenlythiourea (Sigma). We used the following published strains: *Tg(fabp10:RFP, ela3l:EGFP)^gz12^* [34], *Tg(ins:CFP-NTR)^s892^* [35], *Tg(ins:dsRed)^m1018^* [36], *Tg(nkx2.2a(-3.5 kb):GFP)^ia3^* (a.k.a. “*duct:GFP*”) [6], and *Tg(ptf1a:eGFP)^jh1^* [21]. To generate the *Tg(hsp70l:loxP-mCherry-stop-loxP-NICD-P2A-Emerald)* line (a.k.a. *Tg(hs:mCherry)*), *H2B-GFP* was replaced in the plasmid *hsp70l:loxP-mCherry-loxP-H2B-GFP-cryaa:Cerulean* [37] by an in-frame PCR fusion product of NICD [38], P2A [39], and Emerald GFP (Invitrogen, Carlsbad, CA). Transgenesis was achieved as described [40].

### Genetic screen and mapping

An ENU mutagenesis screen was executed as described [20]. Bulk segregant analysis was performed using pooled DNA extracted from 20 WT or *donut* mutant embryos. *donut* mutants were subsequently genotyped using a dCAPS strategy: a PCR product amplified by the primer set: 5′-TCCAG CCCAA ACATT CTTTC and 5′- CGTTT GTGTG GGTTG TATAG ACTCA CCACT TGGAA GAGTT TGCCC TCAGT GGCGG CAGcG was digested with HhaI.

### Protein modeling of *donut/I777R*

Pymol software (Schrödinger) was used to manipulate the PDB model of human IPT1-4 [41].

### Immunohistochemistry and immunofluorescence

Whole mount antibody staining, *in situ* hybridization, and proliferation analysis with EdU were performed as described [20]. Proliferation index was calculated using the number of EdU+ cells divided by the total number of exocrine pancreas nuclei, per section; >5 slices were counted per animal. Significance was assessed using student's t test. Fluorescent *in situ* hybridizations were performed as described [42]. Templates for antisense RNA probes were amplified from embryonic cDNA with the following primers: *met*: CGGAG AGAGA GGGAG GAAG and TAATA CGACT CACTA TAGGG AGACA TTGAT GTCCG TGATG GAG; *hgfa*: TGTGT GCTTG AGAAA GAGAG AGA and TAATA CGACT CACTA TAGGG AGATC GACAA ATTGC CACGA TAA; *hgfb*: AGCCA CTGCA GGGAG ACTAC and TAATA CGACT CACTA TAGGG AGAGG GGTAC CTTTT AGGGT GGA; *furina*: GTGTC GGAGT GGCCT ACAAT and TAATA CGACT CACTA TAGGG AGGGT CTTCA TCCCA GGAGT; *furinb*: TGACC TGGAG AGACA TGCAG and TAATA CGACT CACTA TAGGG AATGC TGGGG GATTT TCTCT. For cytoimmunofluorescence, human embryonic kidney cells (HEK293T) were maintained in DMEM supplemented with 10% FBS. HEK293T cells were plated on polylysine-coated coverslips and transfected with lipofectamine with either murine pBabe-puro-Met^WT^ (Addgene) or site directed mutagenesis-generated pBabe-puro-Met^I776R^. Cells were transfected 48 h before immunostaining: unfixed cells, as well as 4% paraformaldehyde-fixed and 0.1% Triton-permeabilized cells were incubated with indicated antibodies for 2 hours prior to incubation with appropriate fluorescent secondary antibodies.

### Immunoprecipitation and Western blot analysis

TOV112D human ovarian carcinoma cells were obtained from ATCC (Manassas, VA) and cultured using a 1∶1 mixture of MCDB 105 medium and medium 199 supplemented with 15% FBS. Met^WT^ and Met^I776R^ were transfected into TOV112D cells with lipofectamine. Coimmunoprecipitations and protein blot analyses were performed as previously described [43]. The antibodies used for immunoprecipitation, protein blot and immunofluorescence are: anti-Met (25H2) and anti-phosphoMet (Cell Signaling), goat anti-Met (extracellular epitope, R&D Systems), rabbit anti-Met (SP260 – intracellular epitope, Santa Cruz).

### Embryo treatment with pathway specific inhibitors

*Tg(nkx2.2a(-3.5 kb):GFP)^ia3^; Tg(ptf1a:dsRed)^jh1^* embryos, distributed in 12-well plates (20 embryos per well), were incubated with different chemicals from 60 to 76 hpf, during the growth of the pancreatic tail. Chemicals were specific inhibitors of MEK (UO126, at 100 µM), p38MAPK (SB203580, at 100 µM), PI3K (LY294002, at 30 µM), Src kinase (SU6656, at 50 µM) and furin (CMK, at 65 µM).

### Embryo microinjection and cell transplantation

The morpholinos (Gene Tools) used for gene knockdown were described in [12], [44]. For experiments at the blastula stage, mRNAs encoding zebrafish wild-type or L775R Met fused to mCherry were synthesized *in vitro*, by kit (Ambion), and 150 pg of mRNA was injected. Live imaging of embryos was performed 5 hours post-injection. For transplantation, capped *cas/sox32* [29] mRNA was synthesized *in vitro*, and donor *Tg(hsp70l:loxP-mCherry-stop-loxP-NICD-P2A-Emerald)* embryos, which exhibit strong, ubiquitous RFP expression in the absence of Cre recombinase, were injected with 200 pg of *cas* mRNA alone or with 4 ng of *met* morpholino. Cell transplantations were performed as described [42]. When *cas*-injected putative *met* mutant cells were transplanted, donors were genotyped after transplantation.

## Supporting Information

Figure S1 *donut* mutants variably lack muscle tone in pectoral fins. (A–C) 9 dpf *Tg(ptf1a:GFP)* larvae imaged from the dorsal aspect to show fin morphology (arrows). In WT larvae, fins are folded closely against the body (A), while in type 1 (B) and type 2 (C) *donut* mutant larvae, the pectoral fins lack tone, and display an open wing configuration. Bar = 0.5 mm.
(TIF)

Figure S2 Heterozygosity for *donut^s908^* sensitizes embryos injected with *met* morpholino. Clutches resulting from *donut* heterozygote to WT crosses were injected with 1 ng of *metMO* and scored for pancreatic morphology. Embryos presenting type 1 and 2 phenotypes were pooled separately from WT clutchmates, and were retrospectively genotyped. Penetrance of short pancreas was increased from 29.1% in WT embryos to 53.4% in heterozygotes. Significance determined using 2-sided Chi squared test, p = 0.0002.
(TIF)

Figure S3 *furina* and *furinb* expression during pancreatogenesis by whole-mount *in situ* hybridization. Both *furina* and *furinb* are expressed in endoderm (brackets); *furina* is expressed in liver (Li) and pancreas (Pa) at 75 hpf. Dorsal views, anterior to the top.
(TIF)

Figure S4 WT endoderm cannot rescue exocrine tail formation in *hgfMO-*injected larvae. (A–B) 84 hpf *hgfMO-*injected host larvae with a high contribution of transplanted WT endodermal cells (red). Extensive contribution of WT cells to both duct and acinar compartments did not rescue type 1 (A) or type 2 (B) exocrine pancreas tail outgrowth.
(TIF)

## References

[1] Field HA, Ober EA, Roeser T, Stainier DY (2003) Formation of the digestive system in zebrafish. I. Liver morphogenesis. Dev Biol 253: 279–290. 12645931 10.1016/s0012-1606(02)00017-9

[2] Niemann HH, Gherardi E, Bleymuller WM, Heinz DW (2012) Engineered variants of InlB with an additional leucine-rich repeat discriminate between physiologically relevant and packing contacts in crystal structures of the InlB:MET complex. Protein Sci 21: 1528–1539. 22887347 10.1002/pro.2142 PMC3526994

[3] Ward AB, Warga RM, Prince VE (2007) Origin of the zebrafish endocrine and exocrine pancreas. Dev Dyn 236: 1558–1569. 17474119 10.1002/dvdy.21168

[4] Biemar F, Argenton F, Schmidtke R, Epperlein S, Peers B, et al (2001) Pancreas development in zebrafish: early dispersed appearance of endocrine hormone expressing cells and their convergence to form the definitive islet. Dev Biol 230: 189–203. 11161572 10.1006/dbio.2000.0103

[5] Field HA, Dong PD, Beis D, Stainier DY (2003) Formation of the digestive system in zebrafish. II. Pancreas morphogenesis. Dev Biol 261: 197–208. 12941629 10.1016/s0012-1606(03)00308-7

[6] Pauls S, Zecchin E, Tiso N, Bortolussi M, Argenton F (2007) Function and regulation of zebrafish nkx2.2a during development of pancreatic islet and ducts. Dev Biol 304: 875–890. 17335795 10.1016/j.ydbio.2007.01.024

[7] Yee NS, Lorent K, Pack M (2005) Exocrine pancreas development in zebrafish. Dev Biol 284: 84–101. 15963491 10.1016/j.ydbio.2005.04.035

[8] Landsman L, Nijagal A, Whitchurch TJ, Vanderlaan RL, Zimmer WE, et al (2011) Pancreatic mesenchyme regulates epithelial organogenesis throughout development. PLoS Biol 9: e1001143. 21909240 10.1371/journal.pbio.1001143 PMC3167782

[9] Serup P (2012) Signaling pathways regulating murine pancreatic development. Semin Cell Dev Biol 23: 663–672. 22728666 10.1016/j.semcdb.2012.06.004

[10] Gherardi E, Birchmeier W, Birchmeier C, Vande Woude G (2012) Targeting MET in cancer: rationale and progress. Nat Rev Cancer 12: 89–103. 22270953 10.1038/nrc3205

[11] Bladt F, Riethmacher D, Isenmann S, Aguzzi A, Birchmeier C (1995) Essential role for the c-met receptor in the migration of myogenic precursor cells into the limb bud. Nature 376: 768–771. 7651534 10.1038/376768a0

[12] Haines L, Neyt C, Gautier P, Keenan DG, Bryson-Richardson RJ, et al (2004) Met and Hgf signaling controls hypaxial muscle and lateral line development in the zebrafish. Development 131: 4857–4869. 15342468 10.1242/dev.01374

[13] Schmidt C, Bladt F, Goedecke S, Brinkmann V, Zschiesche W, et al (1995) Scatter factor/hepatocyte growth factor is essential for liver development. Nature 373: 699–702. 7854452 10.1038/373699a0

[14] Uehara Y, Minowa O, Mori C, Shiota K, Kuno J, et al (1995) Placental defect and embryonic lethality in mice lacking hepatocyte growth factor/scatter factor. Nature 373: 702–705. 7854453 10.1038/373702a0

[15] Johansson M, Mattsson G, Andersson A, Jansson L, Carlsson PO (2006) Islet endothelial cells and pancreatic beta-cell proliferation: studies in vitro and during pregnancy in adult rats. Endocrinology 147: 2315–2324. 16439446 10.1210/en.2005-0997

[16] Sonnenberg E, Meyer D, Weidner KM, Birchmeier C (1993) Scatter factor/hepatocyte growth factor and its receptor, the c-met tyrosine kinase, can mediate a signal exchange between mesenchyme and epithelia during mouse development. J Cell Biol 123: 223–235. 8408200 10.1083/jcb.123.1.223 PMC2119804

[17] Demirci C, Ernst S, Alvarez-Perez JC, Rosa T, Valle S, et al (2012) Loss of HGF/c-Met signaling in pancreatic beta-cells leads to incomplete maternal beta-cell adaptation and gestational diabetes mellitus. Diabetes 61: 1143–1152. 22427375 10.2337/db11-1154 PMC3331762

[18] Mellado-Gil J, Rosa TC, Demirci C, Gonzalez-Pertusa JA, Velazquez-Garcia S, et al (2011) Disruption of hepatocyte growth factor/c-Met signaling enhances pancreatic beta-cell death and accelerates the onset of diabetes. Diabetes 60: 525–536. 20980460 10.2337/db09-1305 PMC3028352

[19] Aparicio IM, Garcia-Marin LJ, Andreolotti AG, Bodega G, Jensen RT, et al (2003) Hepatocyte growth factor activates several transduction pathways in rat pancreatic acini. Biochim Biophys Acta 1643: 37–46. 14654226 10.1016/j.bbamcr.2003.08.007

[20] Anderson RM, Bosch JA, Goll MG, Hesselson D, Dong PD, et al (2009) Loss of Dnmt1 catalytic activity reveals multiple roles for DNA methylation during pancreas development and regeneration. Dev Biol 334: 213–223. 19631206 10.1016/j.ydbio.2009.07.017 PMC2759669

[21] Godinho L, Mumm JS, Williams PR, Schroeter EH, Koerber A, et al (2005) Targeting of amacrine cell neurites to appropriate synaptic laminae in the developing zebrafish retina. Development 132: 5069–5079. 16258076 10.1242/dev.02075

[22] Basilico C, Arnesano A, Galluzzo M, Comoglio PM, Michieli P (2008) A high affinity hepatocyte growth factor-binding site in the immunoglobulin-like region of Met. J Biol Chem 283: 21267–21277. 18495663 10.1074/jbc.M800727200 PMC2475716

[23] Gherardi E, Youles ME, Miguel RN, Blundell TL, Iamele L, et al (2003) Functional map and domain structure of MET, the product of the c-met protooncogene and receptor for hepatocyte growth factor/scatter factor. Proc Natl Acad Sci U S A 100: 12039–12044. 14528000 10.1073/pnas.2034936100 PMC218709

[24] Birchmeier C, Birchmeier W, Gherardi E, Vande Woude GF (2003) Met, metastasis, motility and more. Nat Rev Mol Cell Biol 4: 915–925. 14685170 10.1038/nrm1261

[25] Komada M, Kitamura N (1993) The cell dissociation and motility triggered by scatter factor/hepatocyte growth factor are mediated through the cytoplasmic domain of the c-Met receptor. Oncogene 8: 2381–2390. 7689722

[26] Mark MR, Lokker NA, Zioncheck TF, Luis EA, Godowski PJ (1992) Expression and characterization of hepatocyte growth factor receptor-IgG fusion proteins. Effects of mutations in the potential proteolytic cleavage site on processing and ligand binding. J Biol Chem 267: 26166–26171. 1334493

[27] Komada M, Hatsuzawa K, Shibamoto S, Ito F, Nakayama K, et al (1993) Proteolytic processing of the hepatocyte growth factor/scatter factor receptor by furin. FEBS Lett 328: 25–29. 8344430 10.1016/0014-5793(93)80958-w

[28] Shapiro J, Sciaky N, Lee J, Bosshart H, Angeletti RH, et al (1997) Localization of endogenous furin in cultured cell lines. J Histochem Cytochem 45: 3–12. 9010463 10.1177/002215549704500102

[29] Kikuchi Y, Agathon A, Alexander J, Thisse C, Waldron S, et al (2001) casanova encodes a novel Sox-related protein necessary and sufficient for early endoderm formation in zebrafish. Genes Dev 15: 1493–1505. 11410530 10.1101/gad.892301 PMC312713

[30] Stafford D, White RJ, Kinkel MD, Linville A, Schilling TF, et al (2006) Retinoids signal directly to zebrafish endoderm to specify insulin-expressing beta-cells. Development 133: 949–956. 16452093 10.1242/dev.02263

[31] Hidalgo M (2012) New insights into pancreatic cancer biology. Ann Oncol 23 Suppl 10: x135–138. 22987949 10.1093/annonc/mds313

[32] Lloyd RV, Hardin H, Montemayor-Garcia C, Rotondo F, Syro LV, et al (2013) Stem cells and cancer stem-like cells in endocrine tissues. Endocr Pathol 24: 1–10. 23435637 10.1007/s12022-013-9235-1

[33] Westerfield M, editor (2000) The zebrafish book. A guide for the laboratory use of zebrafish (Danio rerio). Eugene: Univ. of Oregon Press.

[34] Farooq M, Sulochana KN, Pan X, To J, Sheng D, et al (2008) Histone deacetylase 3 (hdac3) is specifically required for liver development in zebrafish. Dev Biol 317: 336–353. 18367159 10.1016/j.ydbio.2008.02.034

[35] Curado S, Anderson RM, Jungblut B, Mumm J, Schroeter E, et al (2007) Conditional targeted cell ablation in zebrafish: a new tool for regeneration studies. Dev Dyn 236: 1025–1035. 17326133 10.1002/dvdy.21100

[36] Shin CH, Chung WS, Hong SK, Ober EA, Verkade H, et al (2008) Multiple roles for Med12 in vertebrate endoderm development. Dev Biol 317: 467–479. 18394596 10.1016/j.ydbio.2008.02.031 PMC2435012

[37] Hesselson D, Anderson RM, Beinat M, Stainier DY (2009) Distinct populations of quiescent and proliferative pancreatic beta-cells identified by HOTcre mediated labeling. Proc Natl Acad Sci U S A 106: 14896–14901. 19706417 10.1073/pnas.0906348106 PMC2736433

[38] Kikuchi Y, Verkade H, Reiter JF, Kim CH, Chitnis AB, et al (2004) Notch signaling can regulate endoderm formation in zebrafish. Dev Dyn 229: 756–762. 15042699 10.1002/dvdy.10483

[39] Kim JH, Lee SR, Li LH, Park HJ, Park JH, et al (2011) High cleavage efficiency of a 2A peptide derived from porcine teschovirus-1 in human cell lines, zebrafish and mice. PLoS One 6: e18556. 21602908 10.1371/journal.pone.0018556 PMC3084703

[40] Thermes V, Grabher C, Ristoratore F, Bourrat F, Choulika A, et al (2002) I-SceI meganuclease mediates highly efficient transgenesis in fish. Mech Dev 118: 91–98. 12351173 10.1016/s0925-4773(02)00218-6

[41] Gherardi E, Sandin S, Petoukhov MV, Finch J, Youles ME, et al (2006) Structural basis of hepatocyte growth factor/scatter factor and MET signalling. Proc Natl Acad Sci U S A 103: 4046–4051. 16537482 10.1073/pnas.0509040103 PMC1449643

[42] Chung WS, Stainier DY (2008) Intra-endodermal interactions are required for pancreatic beta cell induction. Dev Cell 14: 582–593. 18410733 10.1016/j.devcel.2008.02.012 PMC2396532

[43] Delous M, Hellman NE, Gaude HM, Silbermann F, Le Bivic A, et al (2009) Nephrocystin-1 and nephrocystin-4 are required for epithelial morphogenesis and associate with PALS1/PATJ and Par6. Hum Mol Genet 18: 4711–4723. 19755384 10.1093/hmg/ddp434 PMC2778369

[44] Elsen GE, Choi LY, Prince VE, Ho RK (2009) The autism susceptibility gene met regulates zebrafish cerebellar development and facial motor neuron migration. Dev Biol 335: 78–92. 19732764 10.1016/j.ydbio.2009.08.024 PMC2784935

