# Supplementary figures and images for: Hepatocyte Growth Factor Signaling in Intrapancreatic Ductal Cells Drives Pancreatic Morphogenesis
Source: PLoS Genet. 2013 Jul 25;9(7):e1003650. doi: 10.1371/journal.pgen.1003650 (PMC3723531; doi:10.1371/journal.pgen.1003650)

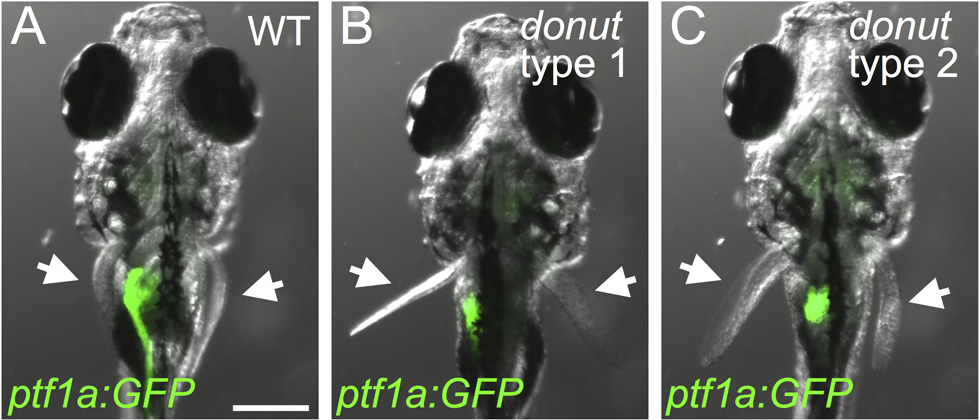

Supplement: Figure S1 — donut mutants variably lack muscle tone in pectoral fins. (A–C) 9 dpf Tg(ptf1a:GFP) larvae imaged from the dorsal aspect to show fin morphology (arrows). In WT larvae, fins are folded closely against the body (A), while in type 1 (B) and type 2 (C) donut mutant larvae, the pectoral fins lack tone, and display an open wing configuration. Bar = 0.5 mm. (TIF) [file pgen.1003650.s001.tif]

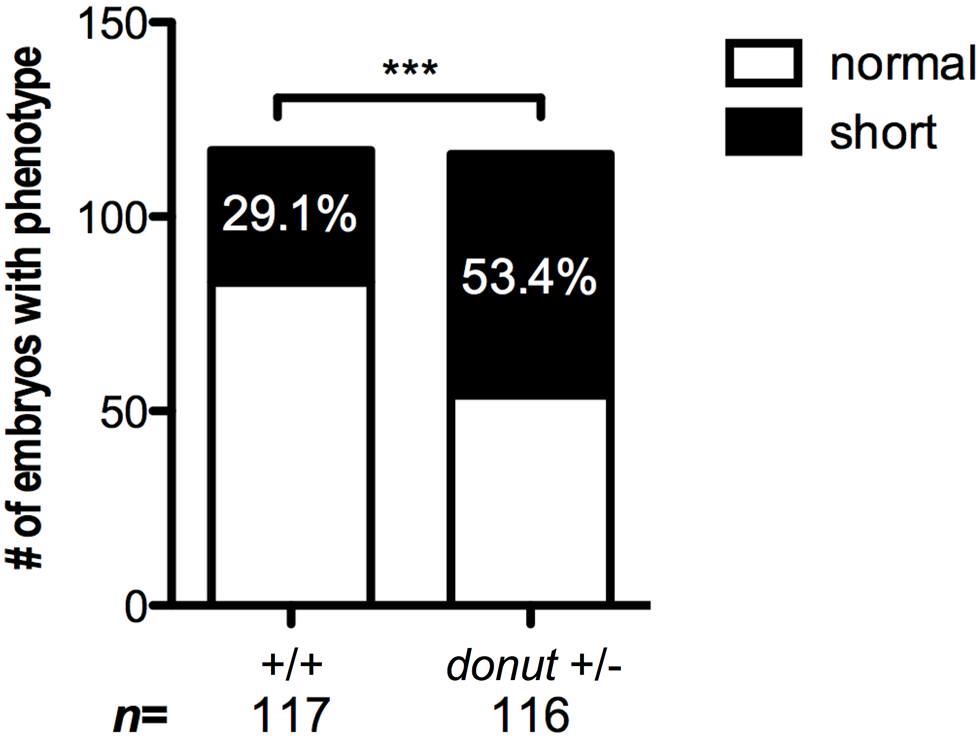

Supplement: Figure S2 — Heterozygosity for donuts908 sensitizes embryos injected with met morpholino. Clutches resulting from donut heterozygote to WT crosses were injected with 1 ng of metMO and scored for pancreatic morphology. Embryos presenting type 1 and 2 phenotypes were pooled separately from WT clutchmates, and were retrospectively genotyped. Penetrance of short pancreas was increased from 29.1% in WT embryos to 53.4% in heterozygotes. Significance determined using 2-sided Chi squared test, p = 0.0002. (TIF) [file pgen.1003650.s002.tif]

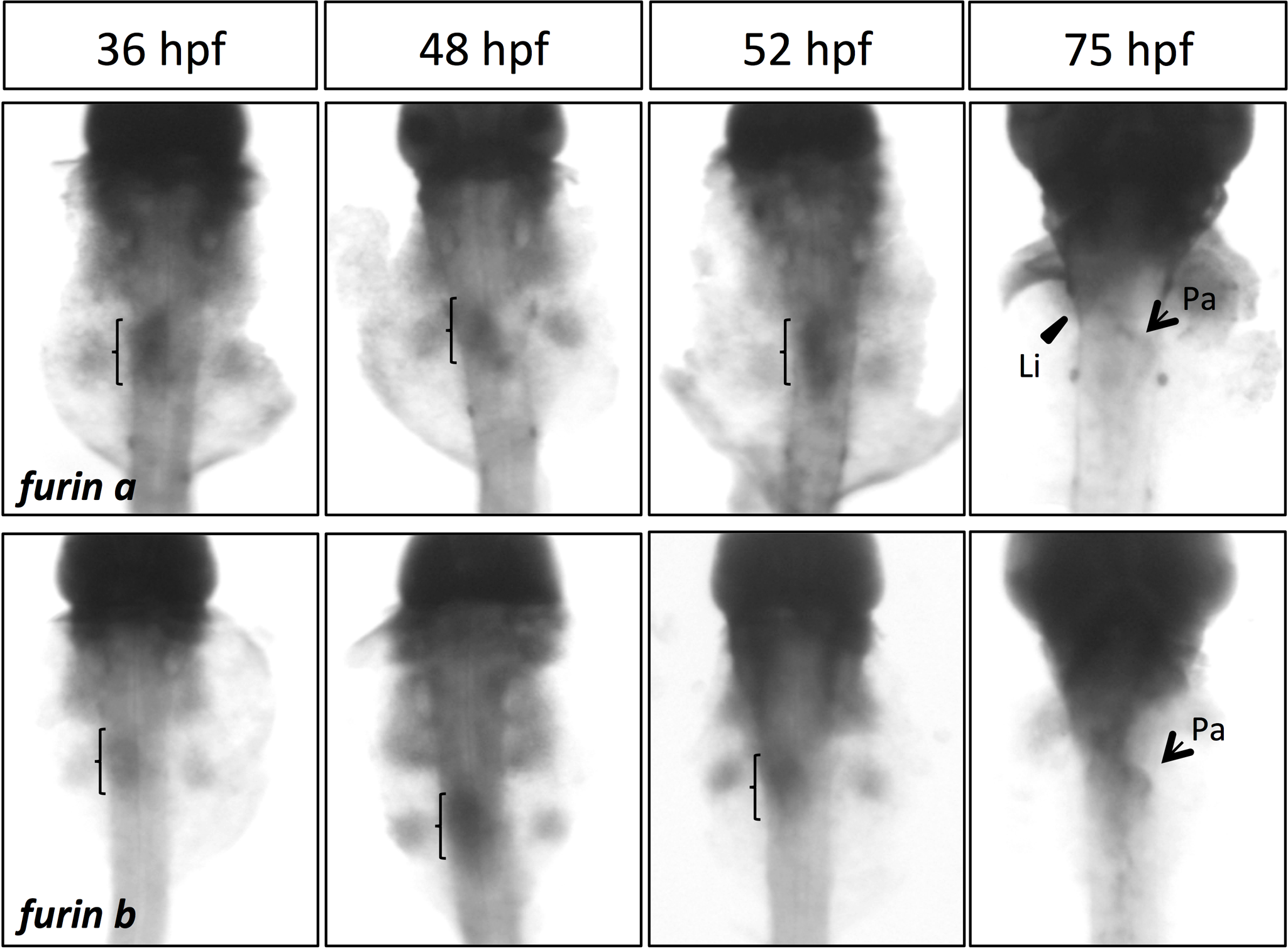

Supplement: Figure S3 — furina and furinb expression during pancreatogenesis by whole-mount in situ hybridization. Both furina and furinb are expressed in endoderm (brackets); furina is expressed in liver (Li) and pancreas (Pa) at 75 hpf. Dorsal views, anterior to the top. (TIF) [file pgen.1003650.s003.tif]

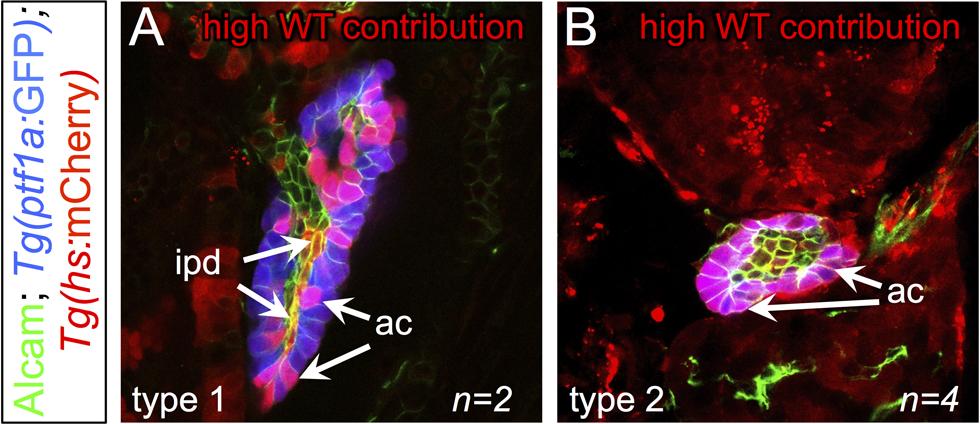

Supplement: Figure S4 — WT endoderm cannot rescue exocrine tail formation in hgfMO-injected larvae. (A–B) 84 hpf hgfMO-injected host larvae with a high contribution of transplanted WT endodermal cells (red). Extensive contribution of WT cells to both duct and acinar compartments did not rescue type 1 (A) or type 2 (B) exocrine pancreas tail outgrowth. (TIF) [file pgen.1003650.s004.tif]
